# GRADE Concept 7: Issues and Insights Linking Guideline Recommendations to Trustworthy Essential Medicine Lists

**DOI:** 10.1016/j.jclinepi.2023.111241

**Published:** 2024-02

**Authors:** Thomas Piggott, Lorenzo Moja, Kristina Jenei, Tamara Kredo, Nicole Skoetz, Rita Banzi, Dario Trapani, Trudy Leong, Michael McCaul, John N. Lavis, Elie A. Akl, Francesco Nonino, Alfonso Iorio, Joanna Laurson-Doube, Benedikt D. Huttner, Holger J. Schünemann

**Affiliations:** a Department of Health Research Methods, Evidence, and Impact, McMaster University, Hamilton, Canada; b Department of Family Medicine, Queens University, Kingston, Canada; c Department of Health Product Policy and Standards, World Health Organization, Geneva, Switzerland; d Department of Health Policy, London School of Economics and Political Science, London, United Kingdom; e Health Systems Research Unit, South African Medical Research Council, Cape Town, South Africa; f Division of Clinical Pharmacology, Department of Medicine and Division of Epidemiology and Biostatistics, Department of Global Health, Faculty of Medicine and Health Sciences, Stellenbosch University, Stellenbosch, South Africa; g Institute of Public Health, Faculty of Medicine and University Hospital Cologne, University of Cologne, Cologne, Germany; h Mario Negri Institute for Pharmacological Research, IRCCS, Milan, Italy; i Department of Oncology and Hematology, University of Milan, Milan, Italy; j European institute of oncology, IRCCS, Milan, Italy; k Division of Epidemiology and Biostatistics, Department of Global Health, Centre for Evidence-Based Health Care, Faculty of Medicine and Health Sciences, Stellenbosch University, Cape Town, South Africa; l McMaster Health Forum, McMaster University, Hamilton, Canada; m Africa Centre for Evidence, University of Johannesburg, Johannesburg, South Africa; n Department of Internal Medicine, American University of Beirut, Beirut, Lebanon; o IRCCS Istituto delle Scienze Neurologiche di Bologna, Unit of Epidemiology and Statistics, Bologna, Italy; p WHO Collaborating Centre in Evidence-Based Research Synthesis and Guideline Development, Regione Emilia-Romagna, Bologna, Italy; q Department of Research Methods, Evidence, and Impact, Mike Gent Chair in Healthcare Research, McMaster University, Hamilton, Canada; r Multiple Sclerosis International Federation, London, United Kingdom; s Department of Biomedical Sciences, Humanitas University, Milan, Italy; t Department of Medicine, McMaster University, Hamilton, Canada

**Keywords:** Essential medicines, Drug coverage, GRADE, Guidelines, Health decision-making, Health policy

## Abstract

**Objectives:**

Guidelines and essential medicine lists (EMLs) bear similarities and differences in the process that lead to decisions. Access to essential medicines is central to achieve universal health coverage. The World Health Organization (WHO) EML has guided prioritization of essential medicines globally for nearly 50 years, and national EMLs (NEMLs) exist in over 130 countries. Guideline and EML decisions, at WHO or national levels, are not always coordinated and aligned. We sought to explore challenges, and potential solutions, for decision-making to support trustworthy medicine selection for EMLs from a Grading of Recommendations, Assessment, Development and Evaluations (GRADE) Working Group perspective. We primarily focus on the WHO EML; however, our findings may be applicable to NEML decisions as well.

**Study Design and Setting:**

We identified key challenges in connecting the EML to health guidelines by involving a broad group of stakeholders and assessing case studies including real applications to the WHO EML, South Africa NEML, and a multiple sclerosis guideline connected to a WHO EML application for multiple sclerosis treatments. To address challenges, we utilized the results of a survey and feedback from the stakeholders, and iteratively met as a project group. We drafted a conceptual framework of challenges and potential solutions. We presented a summary of the results for feedback to all attendees of the GRADE Working Group meetings in November 2022 (approximately 120 people) and in May 2023 (approximately 100 people) before finalizing the framework.

**Results:**

We prioritized issues and insights/solutions that addressed the connections between the EML and health guidelines. Our suggested solutions include early planning alignment of guideline groups and EMLs, considering shared participation to strengthen linkage, further clarity on price/cost considerations, and using explicit shared criteria to make guideline and EML decisions. We also provide recommendations to strengthen the connection between WHO EML and NEMLs including through contextualization methods.

**Conclusion:**

This GRADE concept article, jointly developed by key stakeholders from the guidelines and EMLs field, identified key conceptual issues and potential solutions to support the continued advancement of trustworthy EMLs. Adopting structured decision criteria that can be linked to guideline recommendations bears the potential to advance health equity and gaps in availability of essential medicines within and between countries.

**What is new?**

**Key findings**
• We have found challenges and opportunities in connecting essential medicine lists and health guidelines, as well as addressing opportunities to improve the EML process. We provide insights to improve the connections, use of evidence, consideration of price/cost, consider therapeutic alternatives, contextualize EMLs and consider equity.

**What this adds to what was known?**
• Essential medicine lists and health guidelines address similar topics, our work provides conceptual exploration in a GRADE Concept Paper to strengthen their connections and shared methodology in practice.

**What is the implication and what should change now?**
• Health guidelines and essential medicine lists should engage to more closely aligned processes. Explicit and shared criteria between guidelines and essential medicine lists would strengthen the connection and improve trustworthiness.

**Plain language summary**

• Essential medicines should be available at all times around the world.

• Essential medicine lists pick the most important medicines for the World Health Organization’s (WHO) list and in many countries. In this study we work with experts to explore challenges and ways to make decisions for the essential medicine list (EML) better.

• We propose ways to connect EMLs to guidelines better, topics that could be considered better in EMLs, and connecting EMLs more to lists in different countries to improve these important lists.

## 1 Introduction

Essential medicines are a half-century old concept, with critical modern relevance. Essential medicines should meet priority health needs, be selected based on criteria of public health importance, efficacy, safety, and comparative cost-effectiveness, and are intended to be available at all times in functional health systems [1]. Nearly 50 years ago, in 1977, the World Health Organization (WHO) issued its first essential medicine list (EML) with 168 medicines [1]. The landscape of available medicines has changed dramatically since then, however, the need to prioritize effective medicines that should be accessible to everyone worldwide remains. In fact, this need has achieved renewed interest in recent years with the WHO's commitment to Universal Health Coverage, in the broader context of the United Nation's priority of Universal Health Coverage in Sustainable Development Goal 3.8. In its 23rd iteration, the WHO EML has since now expanded to 502 medicines [2].

Most countries are expected to improve their national coverage by 2030 offering access to essential medicines, however, there are substantial gaps in selection of medicines at the national level compared with those recommended by WHO, specifically for Africa [3]. Over 137 countries have their own national essential medicine lists (NEMLs) [4]. There is wide variability in the number and nature of medicines included in NEMLs compared to those recommended by WHO, which range from only 44 to as many as 983 included medicines [5]. A degree of contextualization would be expected from country to country due to varied epidemiology and health priorities. However, in analyzing national lists by country and therapeutic class there are differences that cannot be explained by factors such as disease prevalence [5]. Additionally, there remain older treatments that have become unsafe or obsolete medicines, that have not yet been removed from the WHO EML, although efforts at keeping up to date have strengthened [6]. Therefore, further work is needed to examine processes and methods to improve transparency and trustworthiness of NEMLs.

Similar to the criticism faced by WHO in the early 2000s, on guideline methodology that did not rigorously incorporate evidence into decision-making, the WHO EML has also been subject to criticism. This criticism has focused on its use of evidence, the composition of the WHO Expert Committee on the selection and use of essential medicines (WHO Expert Committee), the consideration of cost, and the WHO Expert Committee's decision-making processes [[7], [8], [9]]. WHO has made significant progress in the improvement of its guideline methods, based on the Guideline Handbook and advice from its Advisory Committee for Health Research [[10], [11], [12], [13], [14]]. This has included standardization of processes and adoption of methodologies from the Grading of Recommendations, Assessment, Development and Evaluations (GRADE) Working Group [15]. While EMLs are distinct from guidelines, there are many parallels in these paradigms as we have recently demonstrated [16]. We posit that lessons learned from improving trustworthiness of guidelines over the past 2 decades could be applied to improve production of EMLs, in particular for WHO.

Pharmacological interventions are very relevant to the GRADE Working Group, with many health guideline interventions focusing on them. Too often this work does not adequately connect to EMLs and medication coverage lists to ensure implementation of guideline recommendations [16]. While work by the GRADE Working Group has previously examined medicine coverage decisions through the creation of a specific Evidence-to-Decision (EtD) framework tailored to coverage decisions, no previous GRADE guidance has focused on essential medicines [17]. We demonstrated in previous work overlap and synergy in criteria to make decisions for guidelines and EMLs [18]. We also demonstrated usability of an EtD framework for EML decision-making (Piggott, T., unpublished data, 2023).

Criteria in decision-making for the WHO EML are currently derived from the revised procedure for updating WHO's Model List of Essential Drugs, approved by the WHO's Executive Board in 2001 [19]. In the 2023 EML update, the Expert Committee recommended initiating a process to reassess the procedure for updating the EML. As EMLs relate to guideline decisions, this work serves as a starting point for considering conceptual issues with EMLs and their connection to guidelines. This work sits within a larger body of work building toward an EtD framework for EMLs, modeled after the GRADE EtD framework and making decisions in other contexts [16].

## 2 Objective

We sought to explore how the GRADE EtD frameworks and other methodological improvements to guidelines can shed light on challenges, and potentially propose adoption or adaptation of specific criteria that might be useful to address within the WHO EML and with possible applicability to NEML processes.

## 3 Methods

### 3.1 Overview

We followed the process set out by the GRADE Working Group for concept papers, which outlines rigorous methods and policies for the approval of official GRADE articles (Schunemann, H.J., et al., unpublished data) [20]. The development of a GRADE paper is initiated by GRADE project group leads (in this case TP, LM, and TK). The project group leads draft Terms of Reference outlining the role of the group, group leads, GRADE Guidance Group liaison (in this case HJS), the specific objectives of the group, deliverables, and timeline for the work. The approved GRADE for EMLs Terms of Reference are included as appendix 1. We used the GRADE Working Group meetings and case studies to discuss identified key issues on EMLs and guidelines and propose solutions/insights. As a concept paper this presents preliminary concepts that will be further refined by the GRADE EML Project Group and eventually developed into formal guidance.

### 3.2 Identification of key issues and solutions/insights

After extensive preparatory work (as part of TP thesis dissertation), we held an initial, hybrid virtual/in-person project group workshop at the GRADE Working Group meeting on July 11, 2022 in Krakow, Poland (approximately 30 attendees). The purpose was to explore and ultimately establish a link between established GRADE criteria on the EtDs framework and the WHO EML criteria. The project group leads presented key conceptual considerations for EMLs to inform preliminary discussion and priority setting for future GRADE EML project group work. Initial priorities from the workshop were reviewed by project group leadership and used to build a list of key conceptual issues to be included in a survey for the project group members. The purpose of the survey was to priority rank items, while also seeking additional feedback from project group members for new issues and proposals (see Box 1 and appendix 2 for the survey).

Box 1 **Key deliverables from the July 11, 2022 meeting regarding the relation between EMLs and GRADE guidelines**

• How to ensure that comparative cost-effectiveness is taken into account on WHO EML given WHO is not usually a funder of essential medicines and affordability differs by setting?;
• How to approach prioritization work upfront for evidence synthesis for key disease areas/public health needs to inform which medicines should go onto EMLs?;
• How do guideline groups more effectively think about the barriers to availability of and access to essential medicines and what to do about them, so that their guidelines are more useful to inform EMLs?;
• How can EML stakeholders be better engaged/involved early on, perhaps considering the role of shared participants/committee members?;
• How advocacy for medicine availability levers such as tiered pricing or voluntary/compulsory licensing agreements to improve affordability and availability can be advanced through guidelines and EMLs?;
• Explore opportunities to map all guideline recommendations to EML medicines;
• Are guideline groups adequately considering removing redundant or problematic medicines (e.g., antibiotics in context of resistance)?

Key deliverables from the initial project group meeting are shown in box 1 below.

We held a series of online project group meetings, which we recorded and summarized in meeting minutes that were available to project group members. Appendix 2 outlines the results of the prioritization survey conducted to assess the importance and experience in relation to each preliminary challenge identified (Box 1) the characteristics of respondents to our initial survey and the results of prioritization and review of expertise are included in Appendix 2 Table 1 and the rating of importance and experience is provided in Appendix 2 Table 2. The issues identified were iteratively refined into conceptual issues (framed as questions) and corresponding solutions/insights to address them.

### 3.3 Case studies

**Fig. 1:**
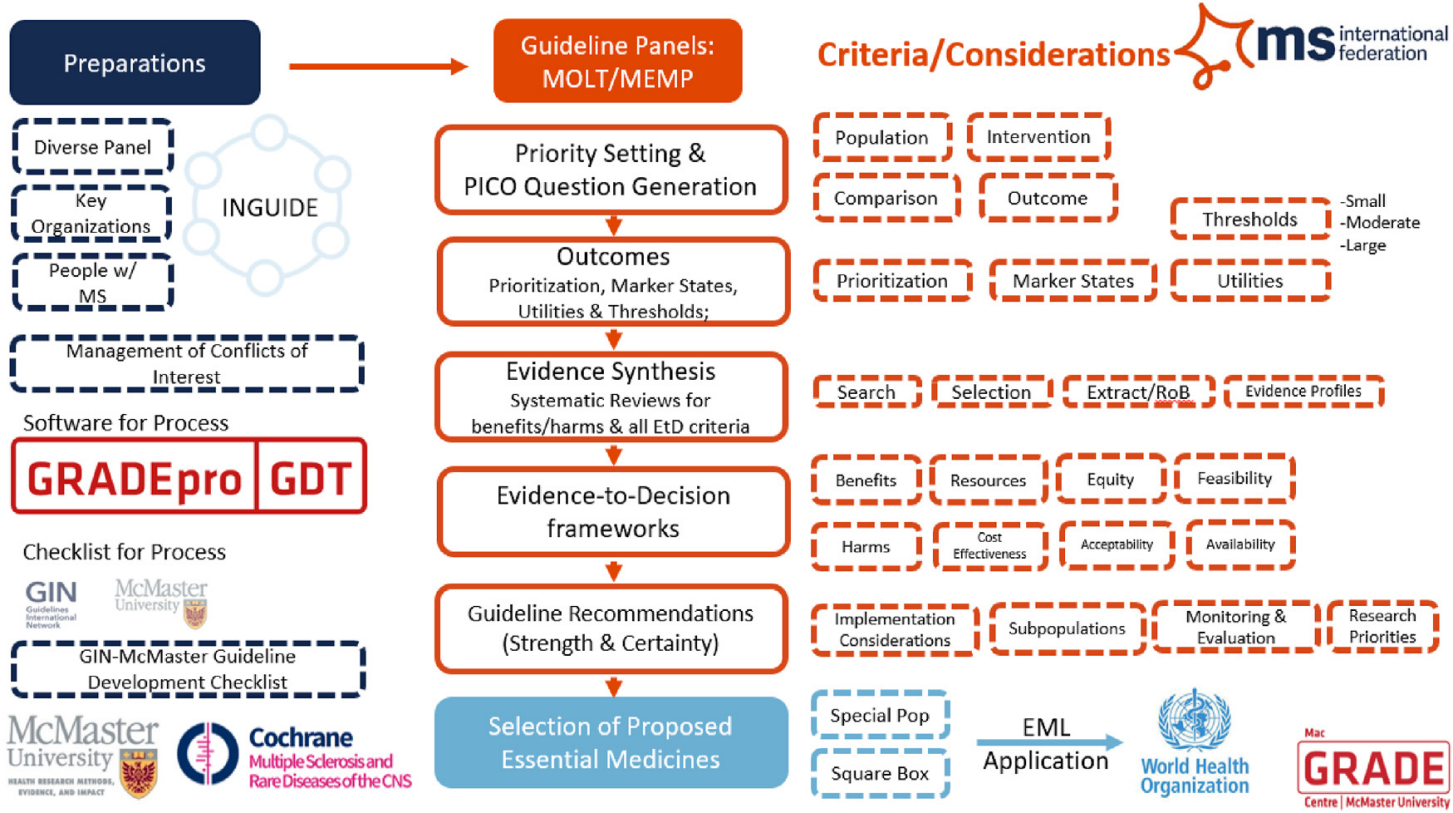
Methods for the linkage between MOLT/MEMP guidelines and an EML application for the treatment of multiple sclerosis. (For interpretation of the references to color in this figure legend, the reader is referred to the Web version of this article.)

**Fig. 2:**
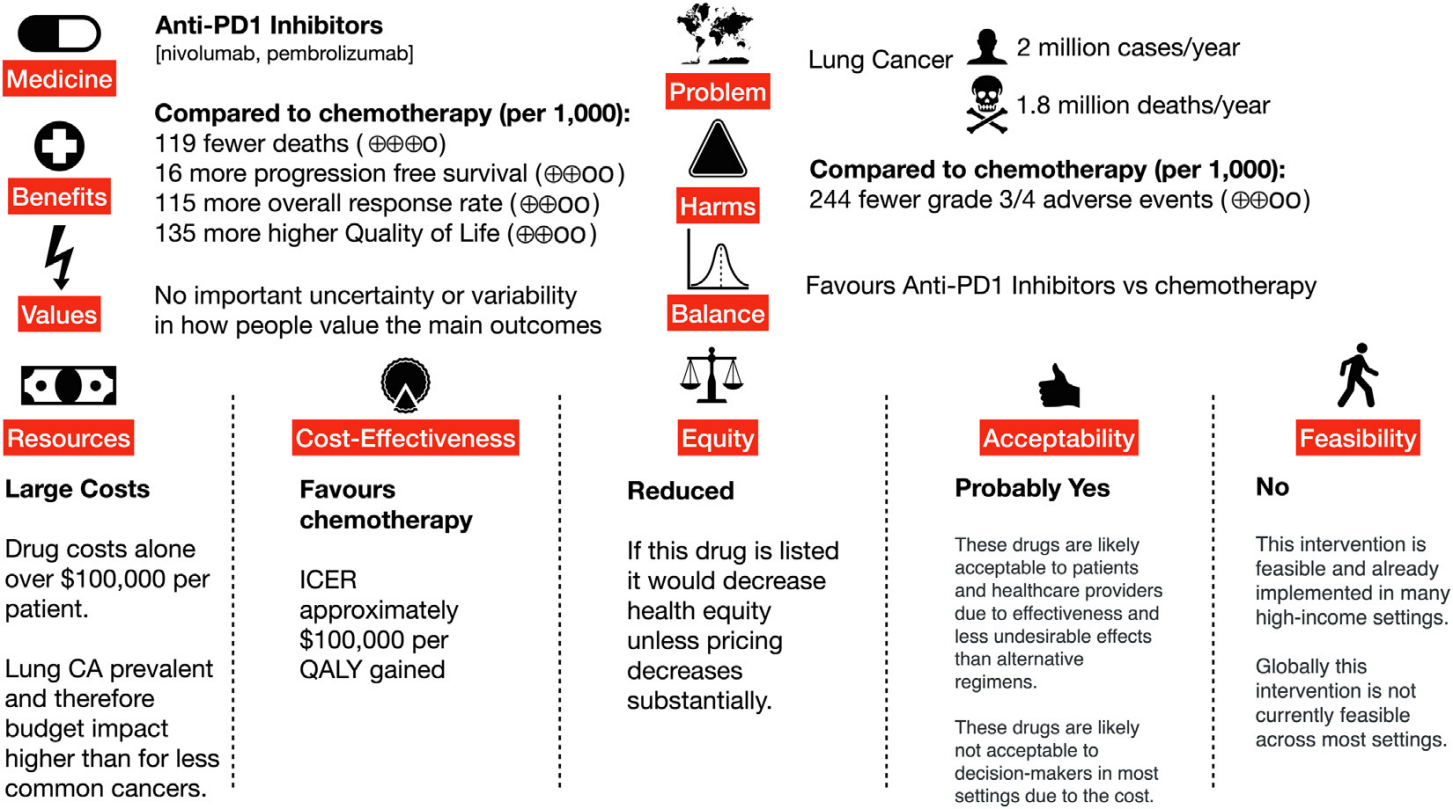
EtD framework decision-criteria visualization. (For interpretation of the references to color in this figure legend, the reader is referred to the Web version of this article.)

**Fig. 3:**
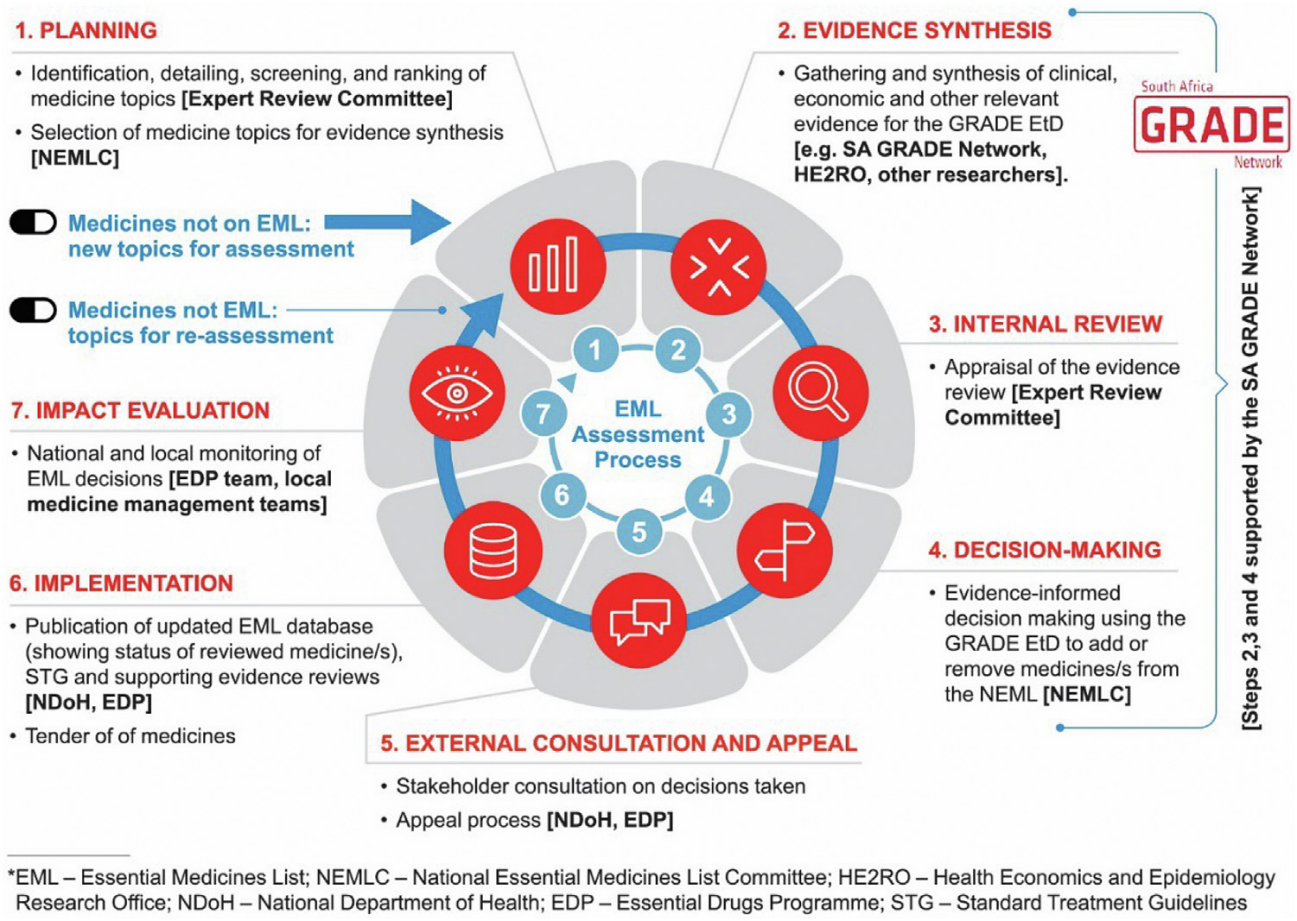
Scope and process for South African Guidelines and EML [36]. (For interpretation of the references to color in this figure legend, the reader is referred to the Web version of this article.)

We prepared and reviewed relevant key case studies (Box 2: Fig 1; Box 3: Fig 2; Box 4: Fig 3). The key case studies identified included: 1) a Multiple Sclerosis International Federation (MISF) guideline effort developed with the expressed purpose of informing an EML application for the 2023 meeting of the WHO Expert Committee; 2) two applications to the 2021 WHO EML–insulin analogs and anti-PD-1/PD-L1 inhibitors for nonsmall cell lung cancer; and 3) the linkage between guidelines and the NEML in South Africa. We iteratively discussed these examples to refine the key conceptual issues, and solutions proposed for them.

Box 2 **Case Study 1: Multiple Sclerosis International Federation (MSIF) Guidelines and 2023 EML Application (Laurson, J., unpublished data) [21]**

The MSIF submitted a 2019 application to the WHO EML for three medicines to treat multiple sclerosis (MS) with support from various stakeholders. The application was rejected, and feedback was provided by the WHO Expert Committee that a comprehensive assessment of all on-label and off-label treatments for MS through a guideline process and subsequent EML application would strengthen the chance of future applications being successful.

Therefore, MSIF worked with Cochrane Multiple Sclerosis and Rare Diseases of the Central Nervous System group and the MacGRADE Center to develop two linked guidelines informed by Cochrane Systematic Reviews and following the GRADE methods for guideline development.

The methods used by MSIF Off Label Treatments panel (MOLT) and MSIF Essential Medicines panel (MEMP) to develop global guidelines for low-resource settings and inform EML application has been described in the publication MSIF guideline methodology for off-label treatments for MS [21].

**The key features of the guideline included**
(a) Protocols, evidence reviews, and final recommendations in peer-reviewed publications.
(b) An international multi-disciplinary panel with members who underwent detailed conflict of interest assessment and management in accordance with the Guideline International Network principles [22].
(c) Cochrane-led systematic evidence collection, synthesis, and assessment using GRADE methodology [23].
(d) Systematic and transparent judgments made by the panel using EtD frameworks [24], standardized terminology for clarity [25] multiple-intervention comparison [26].
(e) Consultation with key stakeholders [21].
(f) Peer-reviewed publications of systematic reviews and guidelines informing this EML application.

Notably, building on previous work this guideline incorporated consideration of availability into the EtD (Piggott, T., et al., unpublished data, 2022) [18]. This included assessing availability information by systematic review reviewing existing medicines on NEMLs, and assessing MS treatment availability data from the MSIF Atlas of MS [27,28]. Prospective cost and availability through follow-on products were also considered by a systematic assessment of the patent landscape across jurisdictions (including low and middle-income countries) by the medicines patent pool [29].

This structured process was found to develop a more rigorous, systematic, transparent, and comprehensive application that was then submitted for consideration in 2023 by the WHO EML Expert Committee.

Box 3 **Case Study 2: Application to the 2021 WHO EML: Anti-PD1/anti-PD-L1 inhibitors for nonsmall cell lung cancer [30]**

A 2021 WHO EML application supported by the European Society for Medical Oncology (ESMO) was considered and was not accepted by the WHO Expert Committee. The application was based on a guideline that was developed in accordance to ESMO methods [31]. These methods involve a review of literature and expert consensus process and uses ESMO guideline methodology and not comprehensive GRADE methods.

To support the linkage between guidelines and the WHO EML a recent project created an EtD framework and one page visualization of decision criteria as adjunctive tools to support the consideration of EML applications by the WHO EML and NEML committee and conducted user-experience testing [32].

A search for existing systematic reviews was undertaken to identify evidence to inform the breadth of decision criteria in GRADE EtDs (benefits, harms, certainty, patients’ values and preferences, balance of effects, resources required, cost-effectiveness, equity, feasibility, acceptability, and availability) in the creation of this tool. Reviews were identified and evidence incorporated for benefits/harms (Cochrane Review), resources required, cost-effectiveness, and availability. No reviews were identified addressing equity, acceptability, or feasibility.

In this process a Cochrane Review on the same Population Intervention Comparison Outcome question as the original application was identified that was published in December 2020, precisely the same month as when the EML application from ESMO was submitted to WHO. This example suggests that a better coordination with other stakeholders engaged in evidence synthesis may have resulted in increased quality, avoiding duplication of work, public confusion, and wastage of limited research resources (e.g., systematic reviewers, guideline developers, health technology assessment, and EML etc.).

Box 4 **Case Study 3: The Connection Between the Standard Treatment Guidelines and Essential Medicines List for South Africa [33]**

South Africa has a two-tiered health system, where the health budget purchases health services for over 85% of the population in the public sector [34]. Rational spend is warranted and thus the ministerially appointed South African National EML Committee considers evidence-informed decision making for selection of essential medicines to the NEML, including aspects of effectiveness, safety, affordability, and feasibility [33]. Furthermore, the South African EML combines both listing of medicines and clinical practice guidelines (CPG) that informs rational use of recommended medicines for primary and secondary level of care in the public sector. Since 2008 the addition of review of medicines to the EML is accompanied by a linked guideline development process. The process is strengthened with grading of evidence evolving from the Strength of Recommendation Taxonomy [35] to the GRADE EtD [24] and from April 2020, 56 medicines on the South African Treatment Guidelines and EML were assessed using the GRADE approach and the EtD Framework, underpinned by efficient rapid reviews to inform rational prescribing of essential medicines. Technical EML Committees are supported by the South Africa-GRADE Network, to conduct reviews utilizing Cochrane Systematic Review methods [36,37] with adaptation/adolopment mechanisms [38] using a systematic step-wise approach, reviewing high quality, up-to-date and relevant CPGs, of randomized controlled trials, randomized controlled trials and then followed by observational studies, as appropriate.

The simultaneous review of the South Africa EML and respective guidelines occurred given resource constraints for evidence syntheses but has since been found to be a superior model to connect guidelines and the NEML, that could be considered elsewhere. Furthermore, the alignment between EMLs and Standard Treatment Guidelines enables implementation of the NEML through more efficient decisions to fund technologies (including medicines) by informing procurement practices using the tender system.

This connection, which involves a combined EML and CPG group, allows for efficient review and synthesis of evidence and a close connection between both processes that are often disparate in other countries.

### 3.4 Concept article preparation and approval

The draft concept paper was presented to the project group and feedback was incorporated, and subsequently presented at the virtual GRADE Working Group in November 2022 (by TP) attended by approximately 120 people. Following the meeting, feedback from the GRADE Working Group was incorporated to develop the final draft concept paper. This was presented and approved on May 24 2023 in Split, Croatia at the GRADE Working Group attended by approximately 100 people and subsequently given final approval by the GRADE Guidance Group before its submission for publication.

## 4 Results

Following extensive project group discussions and feedback, we identify six conceptual issues (framed as questions), and corresponding solutions/insights related to EMLs and their link to guidelines. The results of the prioritization survey are presented in appendix 2. The results highlighted priorities (e.g., key issue #1 connection between systematic reviews, guidelines, and EMLs) and areas where respondents felt they had less expertize (including #3 pathways to access and #6 contextualization of the WHO EML to national level). These provided input for finalization of key issues and solutions/insights discussed by the project group and presented here.

This paper serves as concepts that may be further developed into guidance as additional engagement with key stakeholders continues. Table 1 shows the final list of key conceptual issues, a description of links to examples, and proposed solutions for further exploration and implementation.

**Table 1:** Key conceptual issues for EMLs, examples/evidence, and proposed solutions for further exploration

Key conceptual issues	Examples/evidence	Proposed solutions/insights
1. How can the connection between systematic reviews, guidelines and EML applications be improved to facilitate use of shared evidence syntheses and accelerate access to essential medicines?	The MSIF guidelines (see box 1, MS Off Label Treatments panel (MOLT) and MS Essential Medicines panel (MEMP)) process was conceived to link to an WHO EML application. This resulted in rigorous guidelines and an application to the WHO EML.	1.1 Governance structures tailored to countries (e.g., legislative/legal frameworks) for connecting health decision processes at the country-level should be creating aligning health technology assessments, guidelines, coverage/reimbursement lists and EMLs [16]. 1.2 Shared committee members between guideline and EML committees could provide direct linkage [39]. 1.3 An alignment of Population Intervention Comparison Outcome question priorities between guidelines and EMLs, should be undertaken together on a macro-level (disease categories) and micro (specific medicines) level. 1.4 Requesting that guideline groups consider whether medicines they recommend are essential, and if so prompt linkage to an application to the WHO or NEML. This could be an implementation consideration in established GRADE EtDs (e.g., a section that asks is this medicine current on an EML; if not does the panel feel it should be added through an application?). A link in the EtD to essentialmeds.org or recommendation maps (covid19.recmap.org) could facilitate checking by guideline panels [40]. 1.5 Ensure that EML applicants demonstrate they have reviewed and considered whether medicines they are applying for are supported by health guidelines and for which indications. 1.6 Develop a software solution or streamlined application approach to connect trustworthy guidelines and EML applications (e.g., API (Application Programming Interface) to export evidence from guideline to EML application and vice versa). 1.7 Use of methods already in use in guidelines (e.g., certainty assessment, evidence profiles & evidence-to-decision frameworks) in guidelines informing EMLs or applications to the EML could standardize methods that might improve the trustworthiness of EMLs. The use of a GRADE Evidence-to-Decision framework was recently identified as desirable and feasible for applicants to complete as part of the EML application process [32]. The GRADE EtDs can be used for a variety of decisions, including coverage decisions where GRADE describes five options [17]. Laying out options as binary, listing or not listing, or providing multiples options, e.g., suggesting listing in a national EML under certain conditions such as price negotiations may be required using the GRADE EtDs. 1.8 If the methodological requirements of EML applications are clarified (e.g., requiring a link to systematic review or guideline) an appropriate quality appraisal tool could be used to assess the underlying evidence and quality of applications (e.g., AMSTAR II, ROBIS, AGREE II). This would support choosing the most up-to-date, relevant and credible sources of evidence in the event of multiple eligible systematic reviews or guidelines. 1.9 Established processes to manage conflicts of interest in guidelines could also be used for the management in EML committees (Guideline International Network principles [22]). Feedback from members of the committees using tools such as PANELVIEW may be considered. 1.10 When a guideline (in particular a WHO guideline) makes a recommendation against a medicine that has historically been on essential medicines list for that indication (e.g., evolving evidence demonstrates greater harm than benefit) they should apply to remove that medicine from the EML to ensure coordination between guidelines and EMLs. The opposite is also true: when the EML (in particular the WHO EML) makes a recommendation to not list a medicine as essential that is recommended in a guideline for that indication (e.g., the medicine does not maximize the medical benefit per unit of money spent and not enough funding is available), guideline panels should potentially revise the recommendation.
2. What should the certainty of evidence, strength of recommendation, and key decision criteria (e.g., cost-effectiveness, equity etc.) be for a medicine assessed by a guideline to be considered essential?	Some me-too medicines for cancer face large regulatory hurdles, but could provide more benefits across other EtD criteria such as cost and availability.	2.1 GRADE certainty assessments could be completed for medicines considered by the EML if not available from the source systematic reviews. Similar to GRADE language for informative statements in systematic reviews and guidelines based on the certainty and size of the effect may be useful to communicate key information on EML medicines [41]. 2.2 Absolute effects should be considered by EML applicants and committees, as these take into consideration baseline risk compared to relative effects when making judgments around benefits and harms. Where baseline risk differs substantially, EML committees should considering using contextually appropriate baseline risk to calculate absolute effects. 2.3 Clarity on what weight EML committees should give to outcomes should be sought. It should be explored whether only critical outcomes such as mortality, quality of life should inform decisions for EML committees or whether there are there other important outcomes 2.4 Medicines may be essential even if the evidence on their benefits and harms is low or very low certainty, or a guideline issues a conditional recommendation. This is a fine balance, because you also do not want extensive listing/de-listing of medicines with low or very-low certainty evidence, where the evidence base may evolve. 2.5 The established considerations for strong recommendations with low certainty of evidence may be informative for consideration of listing or not-listing essential medicines that have low or very low evidence [42]: - life threatening situation - uncertain benefit, certain harm - potential equivalence of benefits/harms, clear cost difference - high certainty similar benefits, uncertain harms/cost Unique to the EML, additional criteria may include medicines available to treat a condition with a significant burden of disease or where an important gap in treatment availability exists within the EML. 2.6 EMLs could consider the provisional (or conditional) listing of an essential medicine that has low/very low certainty and recommend additional research. 2.7 Medicines, conditionally recommended by guidelines may be considered for the EML when: - conditional because of variability in values/preferences or cost. - conditional based on baseline risk of a key outcome/burden of disease (e.g., might not be a priority issue for the country [EtD domain 1]). 2.8 Building off recent published work on the decision-criteria for cancer medicines on the WHO EML, clarity should be sought on outcomes important to EMLs and whether disease category specific criteria (such as those provided for cancer) are needed or if the same criteria and thresholds for benefit/harms should apply to all medicines considered and all contexts/disease-settings [43]. 2.9 Use guidance, ideally fully contextualized, including decision thresholds, to support the ranking of medicines to select those that are most essential [44]. 2.10 Consider the range of special populations (e.g., children, pregnant, breastfeeding) that should be covered by selected final medicines that will be proposed to inclusion in an EML.
3. Should availability, price or overall cost of a medicine be considered in whether a medicine should be listed on an EML, or is it in fact an objective of EMLs?	In developing the MSIF MOLT/MEMP guidelines and linked WHO EML application, the guideline group struggled for how to consider issues such as the present state of the availability and price of the medicine, which was found to have large variability between countries, or whether the group should consider a future state where WHO listing facilitates price decreases. This is because the group felt that listing on an WHO EML could help decrease price or align with lowest negotiated medicine price and increase availability and should therefore not be a pre-condition to being considered essential. Drug price is a complex policy landscape where some pharmaceutical companies agree to voluntary licensure from market launch, while other companies may not agree. As a result, drug prices may be dramatically out of proportion compared to production cost. For example, while Trikafta for Cystic Fibrosis has a cost of $325,000 per year, its production cost has been estimated at under $5,000 [45].	3.1 Further explore when price and overall cost should be factors in relation to the benefits/harms balance of a medicine. In some settings this may involve a cost-effectiveness threshold or other willingness to pay threshold (e.g., linked to GDP as presented previously by WHO). However, EMLs consider that price is a fluid and often industry-driven concept. Essential medicines should be made more affordable as much as possible to improve their appropriate use. One such piece of evidence that could inform EML decisions is the range of price negotiated in different countries. This data is frequently not available due to confidentiality agreements, but where there is variability in price due to negotiation and discounts (as much as 80–90% for some medicines), consideration of the lowest price ranges should be considered as feasible in additional settings. Price may be unreasonably high compared to price of production, if there are not alternative solutions this may pose a significant barrier for access to medicines in Low- and Middle-Income country settings [45]. 3.2 Have EML committees clarify whether price or overall cost is in fact a consideration in relation to the WHO Executive Board resolution that states it should not be a reason for not listing a medicine. This stands in contrast to reimbursement lists or coverage decisions, which may have to consider budget impact and affordability of medicines in a country. We discussed that cost as a criteria for WHO EML listing can be like the ‘which came first the chicken and the egg’ situation, because listing on an EML may lead to strategies that decrease market prices (including market concentration, bulk purchasing or voluntary patent agreements). This remains to be assessed empirically. 3.3 Further define how rare diseases should be treated by the WHO and national EML. Budget impact should inform the assessment of inclusion for diseases, which would address rare diseases that may have treatments that could still be considered essential medicines. Guidance on how to use GRADE in rare diseases can be utilized to inform these discussions [[46], [47], [48]]. Examples of guidelines developed using the GRADE methodology and addressing rare disease, rigorously considering issues around price and accessibility can be usefully reviewed [[49], [50], [51], [52]].
4. What approach can be taken to transparently identify therapeutic alternatives (square box indications) for medicines, and how should clinical equivalency be assessed by EML applicants?	In the MSIF MEMP panel (see box 1) a rigorous guideline process included a network meta-analysis of all disease modifying treatments for MS. This included the setting of thresholds based on health state utility values to inform judgments on trivial/small/moderate/large benefits and harms.	4.1 In determining medicines for EMLs, consider medicine groups *a priori* that should be considered as the same therapeutic class and could later be proposed for “square box” symbol listing on the WHO EML. 4.2 In addressing gaps in an essential medicine list, ideally use evidence synthesized by a systematic review and network meta-analysis, or a scoping review, to have a quantitative assessment of the benefits and harms. This may be challenged if there are not randomized control trials for the medicines reviewed. 4.3 Consider established evidence on therapeutic equivalence of medicines (e.g., FDA Orange Book). 4.4 Make use of conceptual guidance on operationalizing biological plausibility in GRADE evidence certainty assessments to inform certainty assessments for me-too medicines being considered by EMLs [53].
5. What can be done to support contextualization of the WHO EML to the national level?	The South Africa EML considers equity in its EtD framework. Through the use of EtD frameworks by the WHO EML this could make synthesis of evidence for new medicines considered by the South Africa EML, and other NEMLs, more efficient and effective.	5.1 A contextualization approach that transparently notes and shares the decision criteria considered for the acceptance or rejection of an essential medicine from the EML to NEMLs, such as GRADE adolopment (or other contextualization tools) could support linking more efficiently to enable changing of criteria as applicable at a local level [38]. 5.2 The WHO EML could provide clear considerations in the form of national implementation considerations that could suggest reasons for countries to consider listing or not listing a newly considered medicine (e.g., epidemiology/problem priority, feasibility, and availability) and suggestions on improving access. 5.3 Future stakeholder engagement work could explore opportunities to harmonize the policies and methods for NEMLs to improve transparency and evidence-based decisions and decrease inappropriate variability in national EMLs. 5.4 Guidance could explore the key local contextualization factors for NEML committees to consider (e.g., local acceptability of listing a medicine may be very important or adjustments in baseline risk of a critical outcome, clinical feasibility, affordability, availability).
6. How should EML committees consider equity?	In the MSIF MOLT/MEMP guideline GRADE guidance on equity, and a systematic review of equity considerations, was used to inform the guideline recommendations and medicines selected for application to the WHO EML. The group considered both within country equity (e.g., medicines that required infusion may decrease equity in rural areas if it is not feasible) and between countries (more expensive medicines may not be affordable in lower income countries and the impact would be negative on global equity). Decreased equity due to high price of medicines was thought of as a modifiable barrier, because the group felt equity could be improved through price reductions.	6.1 EML committees could explicitly include equity as a criteria considered and create a consistent approach for doing so. Currently, the WHO EML implicitly considers equity in many decisions but it is not a criteria outlined in the current procedure (Piggott, T., unpublished data, 2022). 6.2 EML committees could consider GRADE equity guidance to inform equity considerations by EML committees [[54], [55], [56], [57]], this could include consideration of populations outlined by the PROGRESS-Plus and whether they would be positively or negatively impacted by listing a medicine on an EML. 6.3 If equity is an EML criteria, or an assessment of it desired by guidelines linked to EMLs, clarity in instructions needed to focus review on within/between country equity issues, or both. 6.4 Equity in priorities of medicine applications, and guidelines/evidence synthesis work should be considered [58]. Efforts by WHO to facilitate globally equitable prioritization for evidence synthesis should be undertaken.

## 5 Discussion

We developed a GRADE concept article to describe potential solutions for the connection between the WHO EML, NEMLs, and health guidelines. Our proposed solutions and insights will help the ongoing journey to improve the EML. In 2023, the Expert Committee recommended initiating a process to reassess the procedure for updating the EML that will progress in coming years. We begin to address considerations for the Expert Committee.

### 5.1 Strength and limitations

The strengths of this GRADE concept article include the rigorous and expert-engaging process for GRADE papers (Schunemann, H.J., et al., unpublished data). It also includes novel conceptual solutions to key issues related to the development of trustworthy EMLs. The solutions we present advance an important area of research to contribute to better EMLs and ultimately improved access to medicines and universal health care. The key concepts and examples explored in this paper were identified and informed by the project group. It should be noted that although the GRADE for EML project group has expertize primarily focused in evidence synthesis and guideline development, the authorship includes technical officers from WHO working on the EML and other stakeholders. Participants were predominantly from North America, Europe, and South Africa. A limitation, therefore, includes missing other stakeholders’ perspectives, especially those in low-income and middle-income countries where we know there could be wide divergences of national decisions on inclusion of medicines NEMLs [5]. Further engagement of individuals from other global regions should be undertaken to review concepts and unique considerations before GRADE guidance can be developed. To further advance implementation particularly as it relates to reforming governance structures to improve process and alignment of EMLs and guidelines, involvement of appropriate policy-makers and other stakeholders will be needed.

### 5.2 Implications for policy

The WHO EML will soon be 50 years old, and this stands as an opportunity to rethink the EML, criteria to select medicines and its relationship with other WHO normative products such as guidelines. The WHO has faced criticism as described, but also continues to hold important relevance for access to medicines around the world. This work will also inform considerations for a review of the processes [59]. Indeed, this work was presented to a group of experts at a WHO meeting that kicked-off these considerations in November 2023.

Many of our practical solutions can be easily and immediately implemented by guideline groups and EML committees. Organizations that sponsor EML committees, such as the WHO, could consider policy and structural changes that may improve the rigor and trustworthiness of EMLs. Notably, these solutions include early alignment of guideline and EMLs groups since planning phase, potentially considering shared participation to strengthen linkage, using explicit and shared criteria to make guideline recommendations and EML decisions, using specific criteria when deciding to list essential medicines that have low or very low certainty evidence, and consideration of whether new applications for the addition or removal of medicines from EMLs should be made resulting from guideline recommendations. We also provide recommendations to strengthen alignment between the WHO EML and NEMLs, which in part can be done through following principles of guideline contextualization through methods such as GRADE adolopment [16,38]. Finally, we present a range of recommendations for health guidelines to consider EMLs in their planning and development.

Future changes to formal WHO EML decision criteria would require Executive Board discussions and approval by WHO member states. This reform once finalized may apply to NEML committees as well. We note that as compared to many previous applications, the heightened standard we are proposing and effort to link to guidelines may require more expertize, time, and commitment. The alignment with guidelines could reduce duplication of work and overall less effort, so we suggest further assessment of the implications and feasibility of what we are proposing is needed.

### 5.3 Implications for research

Our work presents unanswered questions that should prompt future engagement with additional key stakeholders. These questions include exploration of medicine price-implications and explicit strategies that could be facilitated by listing essential medicines on the WHO EML and NEMLs to decrease price of medicines and improve availability (e.g., tiered pricing, voluntary licensing agreements, tender-based procurement practices, and market concentration). A priority in relation to EMLs for the GRADE Working Group will be to develop a structured and operationalized approach to linking decision criteria for the selection of essential medicines to health recommendations through a GRADE EML EtD. At a country level, further research to understand NEML decision-making process and methods of development across a range of country settings, notably those where inequitable access to medicines is most significant, and whether solutions proposed here have applicability across those settings.

Another area of research includes prioritization of medicines for consideration of inclusion on EMLs. WHO has historically prompted section reviews relating to disease classes. In the most recent relating to cancer medicines, methodological research and sharing to increase transparency [60]. Future research could address how to identify important gaps for diseases, or disease classes to prioritize future EML applications. WHO has created a searchable WHO EML and NEML database [61]. Additionally, an example of connecting health guideline recommendations to the WHO EML is through the eCOVID-19 recommendation map that identifies and links to essential medicines [40]. The availability of additional recommendation maps would improve this connection [62,63]. Thus, this work fits with broader work to identify and advance synergy in a range of health decision-making paradigms, including guidelines and EMLs [16]. We plan to develop a Guideline International Network-McMaster checklist extension for considering EMLs in the guideline development process similar to the extensions for quality assurance and stakeholder engagement [[64], [65], [66], [67]].

## 6 Conclusions

This GRADE concept article based on involvement of key stakeholders from the guidelines and EML development identified key conceptual issues and potential solutions to support the continued advancement of trustworthy EMLs. EMLs are an important prioritization tool, at the global and national-level, that work to prioritize essential medicines to improve their availability and use to improve health in the context of universal health coverage. To advance health equity, gaps in availability of essential medicines should be addressed within and between countries. Our concepts and solutions help taking first steps to achieving this. When additional examples are available, developing a fully operationalized GRADE EML EtD framework may become reality.

## CRediT authorship contribution statement

**Thomas Piggott:** Conceptualization, Data curation, Formal analysis, Investigation, Methodology, Project administration, Resources, Validation, Visualization, Writing – original draft, Writing – review & editing. **Lorenzo Moja:** Conceptualization, Formal analysis, Investigation, Methodology, Validation, Writing – original draft, Writing – review & editing. **Kristina Jenei:** Investigation, Methodology, Writing – review & editing. **Tamara Kredo:** Investigation, Methodology, Validation, Writing – original draft, Writing – review & editing. **Nicole Skoetz:** Investigation, Methodology, Validation, Writing – review & editing. **Rita Banzi:** Investigation, Methodology, Writing – review & editing. **Dario Trapani:** Investigation, Methodology, Validation, Writing – review & editing. **Trudy Leong:** Investigation, Methodology, Validation, Writing – original draft, Writing – review & editing. **Michael McCaul:** Investigation, Methodology, Validation, Writing – original draft, Writing – review & editing. **John N. Lavis:** Investigation, Methodology, Supervision, Validation, Writing – review & editing. **Elie A. Akl:** Investigation, Methodology, Supervision, Validation, Writing – review & editing. **Francesco Nonino:** Investigation, Methodology, Validation, Writing – review & editing. **Alfonso Iorio:** Investigation, Methodology, Validation, Writing – review & editing. **Joanna Laurson-Doube:** Investigation, Methodology, Writing – original draft, Writing – review & editing. **Benedikt Huttner:** Investigation, Methodology, Validation, Writing – review & editing. **Holger J. Schünemann:** Conceptualization, Formal analysis, Investigation, Methodology, Project administration, Resources, Supervision, Validation, Visualization, Writing – original draft, Writing – review & editing.

## Declaration of competing interest

Tamara Kredo is a member of the South African National Essential Medicines List Committee and Standard Treatment Guidelines; Co-lead of South African GRADE Network; Cochrane Board member; Member of the National Advisory Group on Immunisation. Trudy Leong is a member of the SA GRADE Network. Michael McCaul is a Member of the South African Primary Health Care and Adult Hospital Level Expert Review Committee; Founding member of the SA GRADE Network. The remaining authors are all members of the GRADE Working Group, however, have no other interests to declare.

## Data Availability

Data will be made available on request.

## Supplementary data

Appendices 1 and 2
