# Supplementary material for: GRADE Concept 7: Issues and Insights Linking Guideline Recommendations to Trustworthy Essential Medicine Lists
Source: J Clin Epidemiol. 2024 Feb;166:None. doi: 10.1016/j.jclinepi.2023.111241 (PMC10939133; doi:10.1016/j.jclinepi.2023.111241)
Supplement: Appendices 1 and 2 [file mmc1.docx]

## Appendix 1: GRADE for EMLs Project Group Terms of Reference

GRADE project interest group

*GRADE project group on Essential Medicine Lists*

Terms of Reference

| Drafted by: Thomas Piggott  Revised by: Tamara Kredo, Lorenzo Moja, Benedikt Huttner, Holger Schünemann | May 30th, 2022 |
| --- | --- |

**Name of the project group:**

GRADE project group on Essential Medicine Lists,

**Role of the project group**

Essential Medicine Lists (EMLs) are critical for prioritizing medicines around the world and ensuring people have access to them. Essential medicines meet the priority health care needs of the population, and are intended to be available at all times within functioning health systems in adequate amounts, dosage forms, and quality assurance at an affordable price. The World Health Organization (WHO) Model List of Essential Medicines (MLEM) has prioritized medicines since 1977 and over 137 countries around the world also have NEMLs, which inform coverage decisions and ultimately availability of these medicines to those needing them.

Prescribing of medicines that are deemed essential should be grounded and prescribed based on reliable evidence and, when possible, in close alignment with clinical practice guidelines. However, the relationship between essential medicines and guideline recommendations is not always linear. There are cases in which guideline recommendations are developed first and the same evidence base is used to build the case to include a medicine in the NEML. There are other instances in which a medicine becomes essential while there are not yet guideline recommendations available to guide its use in clinical practice. The opposite is when a guideline recommends a medicine but the same medicine is not listed as an essential medicine as it has never been evaluated. In rare cases guideline and essential medicines list might diverge. A paradigmatic case is when a guideline issues a weak recommendation, and the same medicine is rejected as an essential medicine. Weak recommendations might originate legitimate divergences between guidelines and essential medicines lists. However, if a medicine is recommended as part of a strong recommendation, it is more difficult, at least theoretically, to justify a rejection as essential medicine. All these above mentioned scenarios show the complex interplay between two key tools designed to support best care practice.

Despite different approaches for development of guidelines, HTAs, EMLs – the fundamental methods do align in many of the domains that inform decisions/ recommendations. To avoid duplication of efforts, ideally the evidence that informs various health decision products (guidelines, HTAs, EMLs etc) should be based on common methods and rigorous underlying assessment of the research evidence in a format that can be shared (see Schünemann 2022 Lancet Public Health). GRADE has supported Evidence-to-Decision framework develop for a broad range of health decisions (clinical, diagnostic test, public health, coverage decisions etc.). The EtDs support a wide range of health decision-making, and it is believed that the transparency in criteria could support essential medicine selection. This project group will look at issues specific to EMLs as it pertains to not only EtDs but is aimed at exploring the dynamics between guidelines and coverage decisions, in an effort to enhance synergies, improving the trustworthiness and transparency of these complementary tools.

**Specific objectives**

Explore relationship of decision criteria of EML applications to GRADE guidance domains, emphasizing synergistic approaches (e.g. systematic review as foundation evidence supporting the decision making process).

Develop guidance on using methods that reinforce a coordinated approach which encompasses both guidelines and procurement/coverage decisions using GRADE EtDs. Guidance might extend to other prioritarization tools such as the list of essential diagnostics.

Assess what considerations apply primarily to EML applications and what EtD modifications may be required (e.g. addressing availability of medicines, patent and licensing issues, square box and pharmacological equivalency).

Address how applications related to essential medicines can be improved in terms of transparency, comprehensiveness and reporting using GRADE principles.

What do we know about methods for adapting global EML to national settings using GRADE principles?

**Deliverables**

GRADE Concept or Guidance Paper on GRADE for EMLs.

**Timeline**

Year 1 (ending 2022):

- Identify key stakeholders and conduct small group session at the July 2022 GRADE meeting in Krakow.
- Finalize and publish GRADE paper #1 on GRADE for Essential Medicines.

Year 2 (2023):

To be determined.

## Appendix 2: Project Group Priority and Expertise Survey

A prioritization survey was conducted with participants in the GRADE for EMLs project group, n=13 participants (out of approximately 15 regular project group attendees for a response rate of 87%).

| **Appendix 2. Table 1. Respondents’ characteristics of the prioritization survey** | | |
| --- | --- | --- |
| **Characteristic** | **Description** | **Number of Respondents (%)** |
| Area(s) of Expertise | Systematic Review Expert | 9 (69%) |
|  | Health Research Methodologist | 6 (46%) |
|  | EML Committee Member/Technical Expert | 5 (38%) |
|  | Guideline Developer | 4 (31%) |
|  | Health Policy Expert | 2 (15%) |
|  | Health Technology Assessment | 1 (8%) |
| Experience Submitting WHO EML Application(s) | Yes | 7 (54%) |
|  | No | 6 (46%) |
| Experience Submitting National EML Application(s) | Yes | 1 (8%) |
|  | No | 12 (92%) |
| Familiarity with Guideline Development | Median Score, 5-point Likert Scale (Range) | 4 (3-5) |
| Familiarity with EML Development | Median Score, 5-point Likert Scale (Range) | 4 (2-5) |

| **Appendix 2**. Table 2. Prioritization and Identification of Expertise of Preliminary Conceptual Issues | | |
| --- | --- | --- |
| **Key Issue** | **Median Priority, 5-point Likert scale (range)** | **Median Expertise, 5-point Likert scale (range)** |
| 1. How can the connection between systematic reviews, guidelines and EML applications be improved to improve quality and accelerate access? | 5 (2-5) | 3 (3-5) |
| 2. What should the certainty of evidence, strength of recommendation, and key decision criteria (e.g. cost-effectiveness, equity etc.) be for a medicine to be considered essential? (*exception historical use of medicines that will not get new evidence; when there is futility) | 4 (4-5) | 4 (2-5) |
| 3. Should availability of a medicine be considered in whether a medicine should be listed on an EML, or is it an objective of EMLs? | 3 (1-5) | 2 (1-4) |
| 4. What should be considered in determining a pathway to access or implementation plan for essential medicines (e.g. voluntary licensing agreements, market concentration etc)? | 3 (1-5) | 2 (1-4) |
| 5. What approach can be taken to transparently identify square box indications (class effects) for medicines, and how should equivalency be assessed by EML applicants? | 4 (2-5) | 2 (1-4) |
| 6. What can be done to support contextualization of the WHO EML to the National level? | 4 (3-5) | 2 (1-5) |
| 7. How should EML committees consider equity? (framework, e.g. PROGRESS-Plus) | 4 (3-5) | 2 (1-3) |
| 8. How can network meta-analysis and multiple intervention comparison be used to synthesize evidence and support the selection of the most effective essential medicines in a disease area? | 4 (2-5) | 3 (1-5) |
